# Comparative Phylogeography in a Specific and Obligate Pollination Antagonism

**DOI:** 10.1371/journal.pone.0028662

**Published:** 2011-12-27

**Authors:** Anahí Espíndola, Nadir Alvarez

**Affiliations:** 1 Laboratory of Evolutionary Entomology, Institute of Biology, University of Neuchâtel, Neuchâtel, Switzerland; 2 Department of Ecology and Evolution, University of Lausanne, Lausanne, Switzerland; University of Northampton, United Kingdom

## Abstract

In specific and obligate interactions the nature and abundance of a given species can have important effects on the survival and population dynamics of associated organisms. In a phylogeographic framework, we therefore expect that the fates of organisms interacting specifically are also tightly interrelated. Here we investigate such a scenario by analyzing the genetic structures of species interacting in an obligate plant-insect pollination lure-and-trap antagonism, involving *Arum maculatum* (Araceae) and its specific psychodid (Diptera) visitors *Psychoda phalaenoides* and *Psycha grisescens*. Because the interaction is asymmetric (i.e., only the plant depends on the insect), we expect the genetic structure of the plant to be related with the historical pollinator availability, yielding incongruent phylogeographic patterns between the interacting organisms.

Using insect mtDNA sequences and plant AFLP genome fingerprinting, we inferred the large-scale phylogeographies of each species and the distribution of genetic diversities throughout the sampled range, and evaluated the congruence in their respective genetic structures using hierarchical analyses of molecular variances (AMOVA). Because the composition of pollinator species varies in Europe, we also examined its association with the spatial genetic structure of the plant.

Our findings indicate that while the plant presents a spatially well-defined genetic structure, this is not the case in the insects. Patterns of genetic diversities also show dissimilar distributions among the three interacting species. Phylogeographic histories of the plant and its pollinating insects are thus not congruent, a result that would indicate that plant and insect lineages do not share the same glacial and postglacial histories. However, the genetic structure of the plant can, at least partially, be explained by the type of pollinators available at a regional scale. Differences in life-history traits of available pollinators might therefore have influenced the genetic structure of the plant, the dependent organism, in this antagonistic interaction.

## Introduction

The study of both recent (*e.g.*, [1]) and historical (*e.g.*, [2]) species distributional shifts has shown that in a changing environment, organisms follow the cline of their suitable ecological niche. Environmental variations therefore drive changes in distribution ranges. Thanks to a large amount of data available from both geological remains and climatic records, we know that the Quaternary (2.6 – 0 million years ago, Mya) has hosted a series of glacial and interglacial periods, caused by orbital and tectonic events [3], which induced recurrent contractions and expansions of species' distribution ranges [4]. Because such changes impact demographic population parameters, they can affect the genetic variation through processes such as genetic drift and selection [5]. In addition, when contraction processes lead to the fragmentation of a species' distribution area, among-population gene flow might be restricted and lineage divergence could be triggered [4].

The field of phylogeography aims to understand such aspects of the evolutionary history of species, with the assumption that the spatial genetic structure of taxa can be explained by historical and geospatial (*e.g.*, topological, hydrological) features [6]. Phylogeographic patterns of organisms have received considerable attention for the last 20 years, and general post-glacial phylogeographic paradigms have been addressed (see reviews in [7], [8], [9]). In Europe, for instance, most studies have demonstrated that during climate cooling, temperate species found refugia in the southern mountainous European peninsulas (*i.e.*, Balkans, Italy and/or Iberia [9]), and in some cases also in northern areas (*e.g.*, the Carpathians [10]). After the ice retreated, recolonization took place through several routes, with contact zones appearing at natural boundaries such as for example mountain massifs [9].

In contrast to historical and geospatial features, biotic factors have received less attention in the comparative phylogeographic framework, despite they might strongly impact species' survival, dispersal and population dynamics [11] and therefore have the potential to shape spatial genetic structures. In particular, the role of biotic factors in structuring genetic variation should be especially important for species involved in specific and obligate interactions and thus showing high levels of ecological interdependence. Until now, few studies have investigated the comparative phylogeography of species involved in such specific interactions (*e.g.*, the oak gall-wasp and its parasitoids [12], beech and beech parasites [13], aphids and their *Rhus* hosts [14]) and not any has simultaneously analyzed the large-scale variation in spatial genetic structures of both plants and arthropods involved in specific antagonistic interactions at the continental scale.

**Figure 1:**
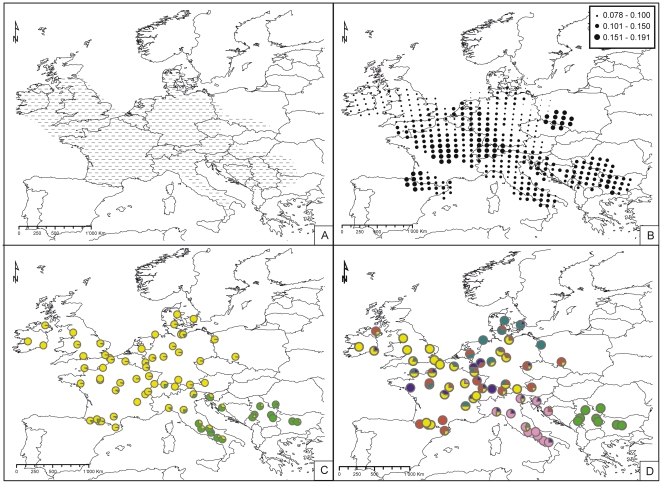
Distribution range of *Arum maculatum* (dashed zone in A; modified from [**17]**, [19****]) and results from spatial genetic analyses (B–D). B) Nei genetic diversities for *Arum maculatum*. C–D) Genetic clusters inferred under model-based (C) and non model-based (D) frameworks. Colours indicate clusters; portions in pie charts indicate probabilities of assignment (model-based) or proportion of individuals (non model-based) assigned to each cluster.

Here, we investigate the comparative phylogeography of a specific and obligate lure-and-trap antagonistic interaction in Europe, involving the Araceae *Arum maculatum* L. and its specific psychodid (Diptera) pollinators *Psychoda phalaenoides* L. and *Psycha grisescens* Tonnoir [15], [16]. In *A. maculatum*, several morphological (*e.g.*, inflorescence shape) and physiological (*e.g.*, odour and heat production, protogyny) adaptations [17] allow the plant to attract ready-to-lay female psychodid flies and trap them during anthesis (a maximum of one day). At the end of this phase, pollen is released and covers the insects, after which they are set free, without receiving any reward. Some of these flies may be trapped a second time by another *Arum* flower, leading to cross-pollination. Experimental studies have furthermore demonstrated that these plants fully rely on insect-vectored cross-pollination for seed production [18]. From a biogeographical point of view, *A. maculatum* is strictly European ([19], [20], Fig. 1A) and the two main pollinators have different distributional ranges. *Psychoda phalaenoides* is distributed worldwide, while *P. grisescens* is found in Europe and in peri-Mediterranean regions [21]. The two fly species do not appear to equally contribute to pollination throughout the plant range; while *P. phalaenoides* is the main pollinator in central and northern Europe, its frequency decreases in southern locations where it is replaced by *P. grisescens* [16].

Due to the asymmetric nature of the interaction (*i.e.*, the plant requires the insects for pollen transfer but the insect is ecologically independent from the plant), two phylogeographic scenarios can be formulated: (i) the genetic structure of the plant is constrained by the presence of its pollinator but not the opposite, a situation that would yield incongruent phylogeographic patterns between the plant and its pollinators (*i.e.*, patterns associated with differences in the distribution of refugia and recolonization pathways); (ii) despite the strong asymmetry in the ecological benefits received by the interacting species, the plant closely follows the pollinators' dispersal during expansion and retraction of available habitats, yielding congruent phylogeographic patterns.

Besides these two contrasting scenarios, according to the theory of the geographic mosaic of coevolution [22] (and considering the marked geographic structure in the respective abundances of the two pollinating species [16]) the genetic structure of the plant could also be associated with insect variation at the inter- rather than intra-specific level. In such a case, biological characteristics (*e.g.*, dispersal capabilities, ecological niche [23]) intrinsic to the most abundant pollinator species in a given region could have driven different regional genetic signatures in the plant through pollinator fitting processes. If this is true, we would expect the genetic structure of the plant to be similar to the spatial variation of the pollinator abundances observed by Espíndola et al. [16].

In this study, we evaluate the phylogeographic scenarios proposed above. Using mitochondrial markers in insects and genome fingerprinting in plants, we first infer the phylogeographies of *A. maculatum* and its two main Psychodid visitors and then quantitatively and qualitatively evaluate their congruence.

## Materials and Methods

### Sampling

Plants and insects were sampled during the springs of 2006–2008, in locations covering the whole geographic range of the plant [17], [20]. Depending of the population size of the visited location, up to 13 plant specimens (minimum two, in one location; Table S1) per population were sampled in 72 locations and dried in silica gel immediately after collection. All flower visitors were preserved in separate Falcon tubes filled with 70% ethanol. Insects could be collected during flower anthesis only, a condition that was fulfilled in 46 locations (Table S1). Insects were identified to the species level following Espíndola *et al.* [16] (Table S1). Considering the sample availability, a maximum of three individuals (minimum one; Table S1) of both *Psychoda phalaenoides* and *Psycha grisescens* were selected from each population for further genetic analyses. No specific permits were required for the described field studies, since the species are not endangered nor protected in the sampled area. Moreover, none of the sampled locations are privately-owned or protected.

### Plant genetic analyses

DNA was extracted using the QIAGEN DNeasy Plant extraction kit, following the manufacturer's protocol (QIAGEN, Hombrechtikon, Switzerland). Because plastid markers in plants are not as variable as in animals [24], gene sequencing would hardly provide any informative data at the intra-specific level. Loci were thus produced with the Amplified Fragment Length Polymorphism (AFLP) technique [25], using *Eco*RI and *Mse*I digestion enzymes, in 362 plant specimens (see below). Further amplification of ligated fragments was performed with two primer pairs (E-ACG/M-CTG and E-AGT/M-CAG). For digestion, 5 µl of DNA were added to get a final volume of 20 µl, containing 1× Buffer 2, 0.1 mg ml^−1^ BSA and 1 unit µl^−1^ of *Mse*I and *Eco*RI (New England Biolabs, Ipswich MA, USA); the mix was incubated at 37°C for 2.00 h. Adaptors were ligated to the digested products at 37°C for 2.00 h, using 40 µl of a mix containing 1× Buffer T4, 0.45 µM adaptor E, 0.36 µM adaptor M, 0.015 units µl^−1^ T4 ligase (Promega SA, Dübendorf, Switzerland) and 20 µl of digestion product. Pre-selective PCR was done in a 20 µl mix composed of 1× buffer GoTaq (Promega SA, Dübendorf, Switzerland), 2 µM MgCl_2_, 250 µM dNTPs (Promega SA, Dübendorf, Switzerland), 0.25 µM primers EA and MC, 0.025 units µl^−1^ *Taq*-polymerase (Promega SA, Dübendorf, Switzerland) and 2 µl ligated DNA. Conditions for this procedure were 2 min at 94°C, followed by 29 cycles of 45 sec at 94°C, 45 sec at 56°C and 1 min at 72°C, and finishing with 10 min final extension at 72°C. The final selective step was done in a 20 µl mix composed of 1× buffer GoTaq (Promega SA, Dübendorf, Switzerland), 2 µM MgCl_2_, 250 µM dNTPs (Promega SA, Dübendorf, Switzerland), 0.4 µM primers EAxx and MCxx, 0.025 units µl^−1^ *Taq*-polymerase (Promega SA, Dübendorf, Switzerland) and 3 µl of a 1/20 solution of the pre-selective PCR product. Selective PCR procedure was 2 min at 94°C, followed by 13 cycles of 30 sec at 94°C, 30 sec at 65°C (with temperature decreasing 0.7°C at each cycle) and 1 min at 72°C, followed by 23 cycles of 30 sec at 94°C, 30 sec at 65°C and 1 min at 72°C, followed by a final extension of 5 min at 72°C. AFLP profiles were genotyped by Macrogen Inc. (South Korea). Allele binning was first automatically processed using Genemapper 3.7 (Applied Biosystems) by applying a threshold of 50 RFU between 50 bp and 350 bp. Bins were then visually checked and adjusted when necessary. Because digestion and amplification problems can arise during the procedure and can highly bias results [26], we randomly chose 36 individuals per plate to be used as intraplate replicates, while five samples were selected out of the total to be used as interplate replicates. The number of bands shared by all replicates of the same sample was examined and repeatability was assessed.

Nei genetic diversities [27] in *Arum maculatum* were calculated for samples having successfully amplified fragments with both AFLP primer pairs, applying a geographic moving window run on R 9.2.1 [28] as in Arrigo *et al.* [29]. The method consists of the following steps: i) the region of study is defined by the most extreme sampled locations, ii) a grid of fixed cell size (in our case 75 km) and their associated centroids are calculated, iii) starting at each centroid a fixed maximum number of samples is randomly selected (three in our case) in a user-defined perimeter (150 km in this study), iv) Nei diversities are calculated for each centroid, v) points iii and iv are repeated 10,000 times, vi) mean values are calculated for each centroid. This method does not only calculates the Nei diversity values at each location, but also shows their variation and distribution throughout the area of study. Moreover, it allows for a correction of putative local sampling biases since the analysis is grid-based. Scripts are available upon request to the first author.

Several methods currently exist for identifying the genetic structure of a molecular dataset [30]. In this study, to identify the most likely number of genetic groups present in our data, we decided to apply two different approaches; model-based and non model-based. In order to identify the genetic structure of *A. maculatum*, 20 runs of 1,000,000 generations with a 200,000 burn-in period for each prior *K* number of populations (set between 1 and 20) were performed using a model-based assignment algorithm as implemented in Structure 2.2 [31]. This method applies Markov chain Monte Carlo (MCMC) searches to identify, given a *K* number of clusters, individual assignment probabilities that optimize Hardy-Weinberg equilibrium at the intra- and inter-cluster levels. Following recommendations of the authors [29], data was coded as diploid and recessive alleles as present. The most probable *K* was identified using the approach proposed by Pritchard *et al.* [32]. A non model-based algorithm was also applied to infer clustering of specimens, by using the *K*-means method, following Hartigan and Wong [33], and selecting for the best number of groups using the inertia criterion (*i.e.*, the average of the distances between the centroid of each cluster and each sample contained in it), following Kergoat & Alvarez [34]. This method, recently applied in a phylogeographic framework by Burnier *et al.* [35] and Arrigo *et al.* [29] is based on the allele composition of each sample and allows the identification of the number of genetic clusters that optimises the grouping of samples. The analysis was performed using R 2.9.1 [28], applying 10,000 replicates for each *K* cluster, for values ranging from 1 to 20. Scripts are available upon request to the first author.

### Insect genetic analyses

DNA from 152 flies (see below) belonging to the two visiting species *P. phalaenoides* (105 samples) and *P. grisescens* (47 samples) was extracted using the QIAGEN DNeasy Blood and Tissue extraction kit (QIAGEN, Hombrechtikon, Switzerland). Psychodids were sampled when trapped in *Arum* flower chambers and were usually covered by pollen. This precluded performing whole genome fingerprinting analyses (such as AFLP) based on total DNA (which has been contaminated with plant DNA from the pollen). Instead, we performed insect-specific single-gene amplification to infer phylogeographic patterns in the insects. We used three mitochondrial regions (hereafter mtDNA), *i.e.*, Cytochrome *b* (Cytb), Cytochrome Oxidase subunit I (COI) and small ribosomal RNA sub-unit (16 s) for *P. phalaenoides* and one, Cytb, for *P. grisescens*. The difference in the number of amplified regions was due to problems encountered during amplification and sequencing in the second fly species, and could not be overcome despite trials under several PCR and mix conditions. PCRs were done in 20 µl of a mix composed of 0.5× buffer, between 1.25 and 2.5 mM MgCl_2_, 10 mM dNTP, 1 unit of GoTaq DNA polymerase (Promega, Dübendorf, Switzerland), 0.5 µM primers (Table 1) and 3 µl DNA and run in a TGradient thermocycler (Biometra, Goettingen, Germany). The program consisted of 2.30 min at 95°C, followed by 35 cycles of 30 sec at 95°C, 40 sec at 57°C or 48.5°C (Table 1), 1 min at 72°C, and finishing with a final elongation of 8 min at 72°C. Amplified fragments were sequenced by Macrogen Inc. (South Korea) and Fasteris SA (Switzerland). Sequences were visually corrected on Chromas Pro 1.41 (Technelysium Pty. Ltd., Brisbane, Australia) and further aligned on BioEdit 7.0.4.1 (Hall 1999). Gaps were coded using the method of Simmons and Ochoterena [36] as implemented in FastGap 1.2 [37].

**Table 1:** Primer sequences and respective annealing temperatures used to sequence portions of insect mtDNA.

Region	Primers	Sequence (5′→3′)	Annealing (°C)
**COI**	C1-J-1718	GGG GGG TTT GGA AAT TGA TTA GTG CC	48.5
	Tl-2-N3014	TCC ATT GCA CTA ATC TGC CAT ATT A	
**Cytb**	CB-J-11338	CAC ATT CAA CCA GAA TGA TAT TT	57
	N1-N-12051	GAT TTT GCT GAA GGT GAA TCA GA	
**16 s**	LR-N-13398	CGC CTG TTT AAC AAA AAC AT	57
	LR-J-12883	CCG GTT TGA ACT CAG ATC ATG T	

Phylogenetic relationships were inferred using Bayesian and Maximum Likelihood (ML) approaches. Models of evolution were estimated using MrAIC v. 1.4.4 [38] and Bayesian analyses were performed in MrBayes 3.1.2 [39], [40] running 50,000,000 generations, with a temperature of 0.5 and sampling one tree every 1,000 generations. ML analyses were run using RaxML 7.2.6 [41], based on 10,000 bootstraps. Complementary phylogenetic networks were constructed for both species using TCS v1.21 [42] applying a 95% connection limit.

Nucleotide diversities were calculated based on Nei genetic diversities using the same procedure as described above for the plant. The same script was used for the two insect species, defining a cell-size of 75 km and a perimeter of 300 km.

Individual-based isolation by distance was investigated with a Mantel test using GenAlex 6 [43]. Pairwise genetic distances between individual samples were calculated using the *Dset* function implemented in PAUP* 4.0 [44], within each species separately, using the models of evolution previously inferred for each partition. This distance calculation allowed us to consider not only the number of variable sites observed in the partition analyzed, but also to adjust distances based on the model of sequence evolution determined for each amplified region. To account for multiple partitions in *P. phalaenoides*, distances were calculated for each partition and afterwards averaged. Geographic distances between samples were calculated and log transformed using GenAlex 6.

### Comparative analysis

The two different methods used to produce molecular data in pychodids and *A. maculatum* (using gene sequencing and genome fingerprinting, respectively) converge in their aim to detect patterns of spatial genetic structure. Comparing their resulting phylogeographic patterns is thus conceptually correct and in agreement with the goals of this study. Spatial genetic structures for the plant and its associated psychodids were displayed on geographical maps using ArcMap 9.3 (ESRI, Redlands CA, USA), with populations represented as pie charts showing the number of individuals assigned to each cluster or haplotype.

Congruence between the genetic structure of plants and insects was evaluated in two different ways. First, it was addressed qualitatively on visual grounds and considered to be supported if the spatial distribution and the number of genetic clusters were roughly similar for both the plant and the insects.

Second, a reciprocal quantitative approach was applied based on an analysis of molecular variance (AMOVA [45]) in both the plant and the insects. This analysis evaluates the level of variation in the genetic diversity at different hierarchical levels, providing an estimate of significance of values through non-parametric permutations. Here we performed AMOVAs for both the plant and insects datasets in order to identify factors that could explain the genetic structure of the studied species. Congruence was assumed if regions significantly explained (based on 10,000 permutations) the molecular variance found in the data. The highest level of hierarchy in a given species was defined by the structure of its associated organism. For the plant, since pollinators did not harbor any spatially defined genetic structure (see Results) the highest level was defined by the two areas where *P. phalaenoides* and *P. grisescens* have been respectively observed to be the main visitors [16]. Only plant locations for which insects were available were considered in this analysis (46/72, see Table S1). For insects, populations were assigned to regions based on the genetic clusters identified in the plant, taking into account both the model and non model-based results (see Table S1 for details on population assignments). The second and third tested levels corresponded to the variation between populations within groups and to the intra-population variation, respectively. All calculations were performed using Arlequin 3.5 [46], with individual-based Nei genetic distances [47] for the plant and individual-based genetic distances obtained through the Paup* 4.0 *Dset* option (see above) for insects. This approach was preferred to other methods because: i) precise dating is impossible in the group due to a lack of fossil data and thus temporal divergence estimations (*e.g.*, [48]) are not appropriate in our framework; ii) strictly dominant data was used in plants, which did not allow the use of coalescent approaches (*e.g.*, [49]).

## Results

### Phylogeography of *A. maculatum*

DNA was extracted from 394 individuals, out of which 362 could be successfully processed using AFLPs. Of these, 62 did not amplify with primer pair E-ACG/M-CTG, while four did not amplify with primer pair E-AGT/M-CAG. Primer pairs E-ACG/M-CTG and E-AGT/M-CAG provided 166 and 160 polymorphic markers, respectively. Reproducibility was 96.3% and 95.7% for E-ACG/M-CTG and E-AGT/M-CAG, respectively, calculated according to Bonin *et al.* [50].

Calculation of Nei diversities indicated that the northern edge of the distribution of *A. maculatum* shows genetic diversities lower than those from southern and central Europe (Fig. 1B).

Structure runs concurred in demonstrating that the most likely *K* number of genetic clusters in *A. maculatum* was two. Genetic groups were geographically structured (Fig. 1C) with one group distributed throughout the Balkans and Italy and the other covering the rest of Europe.

The non-hierarchical *K*-means approach indicated that the *K*-value providing the optimal inertia was six (Figure S1). When the spatial distribution of the six clusters was investigated, four of them appeared highly admixed in the central and northern European region, while the remaining two were clearly differentiated; one in the Balkans and Italy, and the other in the Carpathians (Fig. 1D).

### Phylogeography of *P. phalaenoides* and *P. grisescens*

A total of 105 samples of *P. phalaenoides* successfully amplified at all three regions (1428 bp), while 47 *P. grisescens* amplified at one region (679 bp) (Table 2; sequence accession numbers in Table S2).

**Table 2:** Constant (C), variable (V), parsimony-informative (PI) and total sites per species and sequenced region, in base pairs, for the three mtDNA regions sequenced in insects.

Species	Region	C	V	PI	Total
***P. phalaenoides***	16 s	209	14	0	223
	Cytb	646	34	14	680
	COI	509	16	5	525
	**Total**	1364	64	19	**1428**
***P. grisescens***	Cytb	611	68	13	**679**

**Figure 2:**
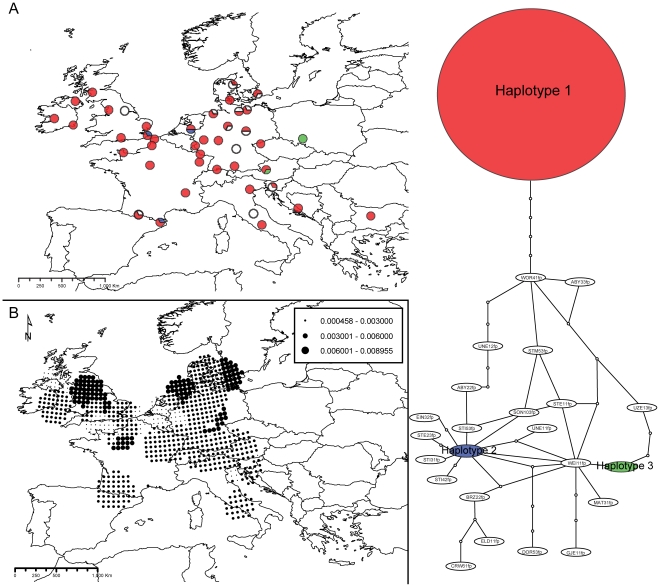
Haplotype network, respective geographic distribution of most-frequent haplotypes (A), and distribution of nucleotide diversities for *Psychoda phalaenoides* (B). Colours and portions of pie charts in A) represent main haplotypes and the proportion of samples assigned to each main haplotype at each location, respectively. Dot sizes in B) are proportional to diversity values (see figure caption).

**Figure 3:**
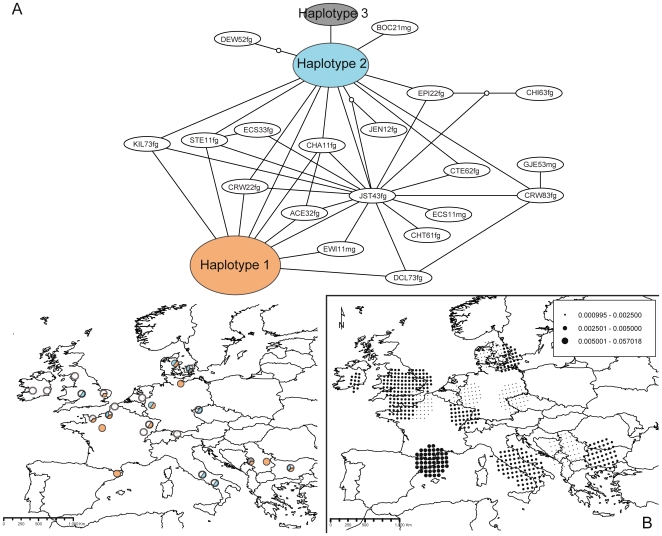
Haplotype network, respective geographic distribution of most-frequent haplotypes (A), and distribution of nucleotide diversities for *Psycha grisescens* (B). Colours and portions of pie charts in A) represent main haplotypes and the proportion of samples assigned to each main haplotype at each location, respectively. Dot sizes in B) are proportional to diversity values (see figure caption).

Both phylogenetic topologies (Figure S2) and haplotype networks (Fig. 2A and 3A) indicated that no geographic structure of the genetic variation was retrieved in either of the two psychodid species analyzed. For convenience of representation, we illustrate this result by focusing on the haplotype network approach. Phylogenies confirmed the monophyly of each fly species, clarifying the fact that *P. phalaenoides* and *P. grisescens* represent well-defined specific entities (an average of around 50 nucleotide differences between the two species, *versus* an average close to 3 at the intraspecific level) that do not hybridize.

In *P. phalaenoides* (Fig. 2A), 24 different haplotypes were recovered, 21 of them unique to single specimens (white in Fig. 2A). The three others (red, blue and green, in Fig. 2A) were present in 80, three and two specimens, respectively. While the most frequent haplotype (red) was separated from all the others by at least four mutational steps, the remaining types were generally more closely related, with a few exceptions separated by a larger number of mutational steps. Even if unique haplotypes appeared to be relatively more abundant in the eastern edge of the sampled zone, two main haplotypes (red and blue) were present homogeneously across the sampling range and the remaining haplotype shared by more than one specimen (green) was present in only two samples at the eastern edge of the distribution. The distribution of nucleotide diversity (Fig. 2B) indicated that main centres of diversity were in northern Europe.

In *P. grisescens* (Fig. 3A), 22 different haplotypes were retrieved, 19 from single specimens (white). The three others (orange, turquoise and grey) were present in 13, nine and three specimens, respectively. All 22 haplotypes were highly interrelated, with 20 of them showing only one step to the closest haplotype and two demonstrating distances of two steps. Here again, the spatial distribution of the main haplotypes was not structured. One main centre of genetic diversity was identified in the Pyrenees region, with north-eastern European zones being less diverse (Fig. 3B).

When testing individual-based isolation by distance (Figure S3), results indicated that in both species there was no correlation between log-transformed genetic and geographic distances (R^2^ = 0.002 and 0.006 for *P. phalaenoides* and *P. grisescens*, respectively, with *P* values>0.05 in both cases).

### Comparison of phylogeographic patterns

The phylogeographic patterns of *A. maculatum* and each of the two psychodids presented no visual congruence: neither the number nor the distribution of genetic groups was similar between the interacting species. While the plant showed a trend towards a geographically-driven structure of its genetic variation (Figs. 1C and 1D), insect haplotypes were distributed without regard to any spatial signal (Figs. 2 and 3).

The hierarchical AMOVA performed on the plant dataset showed that the pollinator compositions provided significant values of Φ-statistics at all levels (Table 3). Φ_ST_ values (0.813) were, however, higher than both Φ_SC_ (0.158) and Φ_CT_ (0.03) values. While 81% of the variation was explained at the intra-population level, only 16% and 3% were found at the inter-population and among-groups levels, respectively.

**Table 3:** Analysis of Molecular Variance (AMOVA) in the three interacting species.

A)					
	Variance component	variance	% variation	Φ-stat	*P*-value
***Arum***	Among groups	0.37812	2.97	Φ_CT_ 0.030	<0.0001
***maculatum***	Among populations within groups	1.94906	15.31	Φ_SC_ 0.158	<0.0001
	Within populations	10.40237	81.72	Φ_ST_ 0.183	<0.0001

B)					
	Variance component	variance	% variation	Φ-stat	*P*-value
***Psycha***	Among groups	−0.123/−0.618	−3.54/−18.18	Φ_CT_ −0.035/−0.182	0.308/0.184
***grisescens***	Among populations within groups	1.401/1.741	40.34/51.20	Φ_SC_ 0.389/0.433	0.197/0.291
	Within populations	2.196/2.278	63.2/66.98	Φ_ST_ 0.368/0.330	0.128/0.863
***Psychoda***	Among groups	−0.044/−0.022	−3.7/−1.81	Φ_CT_ −0.037/−0.018	0.977/0.892
***phalaenoides***	Among populations within groups	−0.045/−0.035	−3.77/−2.86	Φ_SC_ −0.036/−0.281	0.774/0.715
	Within populations	1.273/1.273	107.47/104.68	Φ_ST_ −0.075/−0.047	0.855/0.861

Variance, percentage of variation, Φ-statistics and *P*-values are shown.

A) AMOVA for the plant, based on the structure of visitors' composition observed by Espíndola *et al.* [16]: two groups were defined as a function of the main pollinator (either *P. phalaenoides* or *P. grisescens*). B) AMOVA for insects, based on the spatial genetic structure of the plant. First and second values correspond to results obtained when considering the model- (K = 2) and non model-based (K = 6) spatial genetic structures of the plant, respectively.

For flies, no structure was identified at any of the three tested levels (*i.e.*, Φ_ST_, Φ_SC_ and Φ_CT_), using both model- (K = 2) or non model- (K = 6) based plant scenarios [see above and Table 3]. Φ-statistics values and percentages of variance were not significant, and were even sometimes negative (an indication of strong lack of structure [46]).

## Discussion

### Spatial genetic structure of the plant

Our results show that *A. maculatum* has a spatially well-defined genetic structure (Fig. 1), with populations from south-eastern Europe showing either one (Structure model-based approach, Fig. 1C) or two (*K*-means non model-based approach, Fig. 1D) *K* clusters, dividing the region into an eastern and a western zone. This pattern is similar to those found in other European organisms, such as *Ursus arctos* and *Sorex araneus* [7], in which one widespread European gene pool was found to be segregated from one or two clusters endemic to south-eastern Europe. The observed division of the south-eastern region into two groups (*i.e.*, Italy and Balkans on the one hand, and Carpathians on the other) with the non model-based approach is also consistent with climatologic records from the Last Glacial Maximum (LGM; around 21–18 Kya). During this period, lowered sea levels in the northern Adriatic resulted in land bridges that enabled communication between the eastern and western coasts of the Adriatic [51], while gene flow with the Carpathians was probably more restricted.

It is worth noting that a large part of the genetic variation identified by the non model-based approach (*i.e.*, four among six clusters) seems to have little spatial structure. However, because the model-based approach did not identify these groups, it is difficult to conclude whether or not the admixed pattern at the central and northern part of the European distribution area is an artefact. The *K*-means approach aims to minimize among-sample distances, a principle that can lead to over-split results: the more clusters are parameterized, the smaller the distances between the centroid of each cluster and each included sample becomes, leading to a bias similar to that of overfitted correlations. Following this hypothesis, the multiple central-European clusters identified would be more likely an indication of the difficulty to define a best *K* using non model-based approaches than a biological reality. This might be supported by the fact that analyses of pairwise Nei genetic distances between the six clusters (data not shown) indicated that those belonging to the Balkans/Italy and the Carpathians presented higher genetic distances to all other clusters, while the pairwise distances among the remaining four were lower.

Nonetheless, if we assume that this result reflects a biological reality, specimens from this region might be carrying a large amount of genetic diversity that was present in pre-LGM populations of *A. maculatum*. In this scenario, plants from the different genetic clusters identified in the *K*-means approach would have, at some time, shared Pleistocene refugia, and among-lineage cross-reproduction might have occurred. The pattern currently observed would thus be a consequence of ancient polymorphism combined with massive gene flow among lineages. After the last glacial retraction (around 13 Kya [52]), immigrants from these refugia would have reinvaded the region, carrying in their genomes both the signature of ancient polymorphisms, but also the effect of more recent refugial genetic admixture. A result that supports this view is the pattern observed in the distribution of Nei diversities (Fig. 1B), which identified centres of high diversity in both Italian and south-eastern areas and in regions where several of these central-European clusters are shown to be admixed.

Another biological explanation could be that pollen transfer mediated by insects has shaped the distribution of the spatial genetic structure observed. If this is true, what we currently observe would be a consequence of an intra-regional genetic admixture, due to post-glacial dispersion guided by pollen transfer. In such a situation, the biology of the main pollinator from the central and northern regions (*i.e.*, *P. phalaenoides* [16]), which is distributed worldwide and thus potentially shows higher dispersion capabilities (particularly into cold regions) than *P. grisescens* (restricted to the warm and dry regions of Europe and the peri-Mediterranean area [16]) might have contributed to the high admixture observed here.

### Spatial genetic structure in insects

Despite the fact that the markers used allowed the identification of a high level of genetic variability in psychodids (more than 20 haplotypes revealed in both cases; Table 2, Fig. 2 and 3), no geographic structure could be retrieved in either of the two species. Considering the large among-population distances (up to 3,800 km between the two most distant sampled locations), this pattern could indicate that gene flow between extremely distant populations is possible in psychodids despite the flies' small sizes. This is in agreement with the significant lack of isolation by distance (Figure S3). Moreover, because of their large population sizes and high levels of multivoltinism (*i.e.*, both species reproduce several times per year), it is likely that substitution rates per year are high (see below). Although it is empirically difficult to quantify dispersal by a direct approach in these species, *P. phalaenoides* was recently sampled in aerial netting surveys in England where insects were collected at around 200 m above ground; a height at which any insect found is considered to be engaged in migratory –and thus potentially large-scale– movement [53]. Moreover, in at least one previous study [54], psychodids were shown to be able to perform extended flights of at least 160 km, with members of this family collected alive from boats navigating at this distance offshore in the North Sea. Such biological evidence is compatible with the absence of spatial genetic structure observed in our study. It is also interesting to point out that high levels of gene flow have also been observed in other multivoltine insect species, as in *Ips typographus* (Coleoptera: Scolytidae) at a regional scale in Switzerland [55].

Another explanation for this lack of phylogeographic structure in combination with high dispersal rates could also be found in the biology of these species, which may have been associated with human activities since the Holocene. It is known that these insects are related to agricultural and other anthropic environments, since they develop in rich substrates as cow dung, sewage works and decaying matter [56], [57]. Because of this characteristic, it is possible that a part of the mixture of genetic types could be a result of both past and recent human activities, such as motorized long distance movements currently used for the transport of cattle and waste. Such an effect of human-mediated dispersal on the spatial genetic pattern of insect species has already been observed in other taxa associated with human activities, such as the bean pest beetle *Acanthoscelides obvelatus* [58].

Despite both species demonstrate similar numbers of haplotypes at the continental scale, the nucleotide diversity analysis showed that zones of high diversity were distributed differently in the two species. While *Psychoda phalaenoides* was especially diverse in northern areas (Fig. 2B), *Psycha grisescens* was so in the south-western area (Fig. 3B). This result suggests differences in dispersion capabilities of the two fly species: while *P. phalaenoides* seems able to carry large amounts of genetic diversity towards its colonization front (probably mediated by frequent large-scale gene flow), *P. grisescens* shows low diversity levels at its northern colonization edge, a pattern likely to be associated with lower dispersal capacities. Differences in the spatial distribution of diversity centres in both species might also indicate that these two psychodids have contrasting ecological niches, which could cause differences in local fitness, directly impacting population sizes and the output of drift. Differences in ecological niches –notably those related to low temperature tolerance and humidity– could moreover also explain that while *P. phalaenoides* is able to disperse to higher latitudes, *P. grisescens* is more restricted to southern, drier and warmer European zones. This point might be addressed in the future by performing ecological niche modelling for the two taxa.

### Comparative Phylogeography in a specific lure-and-trap pollination interaction

The respective presence and absence of genetic structure in *Arum maculatum* and its associated psychodids indicates that there is no congruence among the phylogeographies of the three species. Not finding any evidence of shared spatial genetic structure (Figs. 1C–D, 2A, 3A) nor of similarities in the geographic distribution of genetic diversities (Figs. 1B, 2B, 3B) favours our first hypothesis: because of the unbalanced biological dependence of the species, phylogeographic patterns are different.

It is worth noting that *A. maculatum* displays several costly physiological adaptations (*e.g.*, specific odour and heat production, inflorescence morphology, phenology [18], [59], [60], [61]) to attract and capture insects. Such an array of specific, costly traits suggests that this association has been in place for quite a long time and that the incongruent patterns retrieved here cannot be explained by a putative recent (e.g., post-glacial) origin of the interaction. This is also supported by the high level of specificity in the relationship between the plant and its pollinators throughout the plant's whole distribution range (*i.e.*, *A. maculatum* interacts with only two species, in a spatially structured manner [16]) suggesting that despite being present over a large area and in contact with different environmental conditions and insect communities, the specificity of the interaction is preserved. It is, therefore, likely that the antagonistic interaction has persisted through Pleistocene climatic oscillations even if lineages of the independent insect species did not systematically find shelter in the same refugia as the dependent species (*i.e.*, the plant). However, the maintenance of the interaction and the survival of the dependent plant species (assuming it harbours a low level of physiological plasticity that does not permit reproduction without interacting with psychodids), requires that at least one refugium is shared between *A. maculatum* and its associated psychodids. Similarly, the interaction can persist despite differences in recolonization routes, as long as the independent species are frequent enough to allow the dependent species to survive in locations where suitable abiotic and biotic factors overlap. This appears to be a reasonable hypothesis if we consider that these insects are abundant and widespread in Europe [21], which increases the probability of association between a plant and an adequate partner in virtually any location.

It should be noted that a recent post-glacial recolonization simulation-based study performed by Borer *et al.* [62] proposed that a pattern similar to the one obtained in the present study (*i.e.*, evidence of spatial genetic structure in one interacting species and absence in the other) could be retrieved when the two interacting species disperse at very different rates. This could be the case in the interaction studied here as well, since it is known that psychodid flies can reach enormous population sizes (several thousand individuals per square meter [63]) with extremely short generation times (12–18 generations per year in the flies [57] *vs.* one generation every 10–15 years in the plant [17]). This allows for higher dispersal rates through time, increases the mutation rate per year and enhances the probability of genotype admixture in the case of the insects.

In spite of this incongruence in the spatial genetic structures, the AMOVA performed on the plant dataset significantly showed that a part of the plant molecular variance (3%; Table 3) could be significantly explained by the species identity of its main pollinators, which provides support to our third hypothesis. AMOVAs allow evaluation and testing of the proportions of the molecular variance explained by *a priori* defined grouping factors [45]. However, they have rarely been used in the field of comparative phylogeography, with the exception of a study on the *Yucca* - *Yucca* moth interaction, for which molecular variance of a given species was explained by that of another species with levels ranging from 2.5% to 17% [64]. Our result for the proportion of the plant genetic structure explained by pollinator species composition (*i.e.*, 3%) is thus rather at the lower margin of the results found for the *Yucca*-*Yucca* moth interaction. Still, one should keep in mind that since the *A. maculatum* - psychodid interaction is antagonistic, in contrast to the mutualistic *Yucca* - *Yucca* moth interaction, results from this and our study are hardly comparable. Our AMOVA results should thus be considered as an innovative first examination of the ecological factors explaining the respective genetic structure of antagonistic organisms. They particularly show that pollinators have and/or have had some effect on the phylogeographic history of the plant. If the correlation proposed by our AMOVA is causal, it would indicate that the spatial genetic structure of *A. maculatum* is being shaped by the variation in the biological characteristics (*e.g.*, dispersal abilities, ecological niches) of psychodid pollinators. This relates to one of the principles of the geographic mosaic theory of coevolution [22]. For instance, *P. grisescens* seems more restricted to a narrowly defined region, and thus appears as a less mobile or more ecologically selective species than *P. phalaenoides*, a result that is paralleled by the diversity analyses (Figs. 2B and 3B). Another explanation for differences in structure and diversity patterns might be that during glacial periods, each insect species was independently sharing refugia with different plant lineages. For example, it could be proposed that while *P. phalaenoides* was mainly restricted to the western part of the glacial plant distribution, *P. grisescens* was more abundant in the south-eastern part, allowing thus some adaptation to each of the two currently observed plant lineages. During this period, each of the plant lineages could have evolved to adapt to the most frequent insect present in the area. The correlation we currently observe between the plant spatial genetic structure and the respective distributions of the two insect species would thus be a consequence of a recent coevolutionary process. Due to the fact that this interaction involves physiological features such as odour composition, which are key for the specific pollinator attraction, investigating the volatile compounds found in the different plant lineages could further clarify this point.

In this study we show that, according to one of our working hypotheses, the phylogeographies of specific antagonists are incongruent. Reasons for such a pattern may be explained by the asymmetric ecological dependence between the interacting organisms. Our results however suggest that a part of the genetic structure of the dependent organism (*i.e.*, in our case, the plant) can be explained by the spatial visitation pattern of the most frequently lured pollinator, indicating a possible role played by some of the ecological characteristics of the independent partners in shaping the phylogeography of its dependent associated species.

## Supporting Information

Figure S1 **Inertia computed following Kergoat & Alvarez** [**34**] **, for each K value in the K-means computations on the plant AFLP dataset.**
(DOC)

Figure S2 **Phylogeny of the two Psychodid species inferred applying Bayesian and Maximum Likelihood approaches.** Support values are shown on branches, Bayesian/Maximum Likelihood.
(DOC)

Figure S3 **Isolation by distance for A)** ***Psychoda phalaenoides*** **and B)** ***Psycha grisescens*** **represented by the correlation between log geographic distances and genetic distances.** Regression lines are shown. R^2^ are 0.002 and 0.006 for *P. phalaenoides* and *P. grisescens*, respectively, with *P* values>0.05 in both cases.
(DOC)

Table S1 **Sampled locations with geographic coordinates.** Number of plants, *Psychoda phalaenoides* and *Psycha grisescens* analyzed at each sampled location are given. Assignments to groups for the AMOVA tests are indicated under “group AMOVA”.
(DOC)

Table S2 GenBank accession numbers and TreeBase link to data (**; URL: http://purl.org/phylo/treebase/phylows/study/TB2:S12057 **) of sequences used in this work.**
(DOC)
