# Supplementary figures and images for: Comparative Phylogeography in a Specific and Obligate Pollination Antagonism
Source: PLoS One. 2011 Dec 27;6(12):e28662. doi: 10.1371/journal.pone.0028662 (PMC3246438; doi:10.1371/journal.pone.0028662)

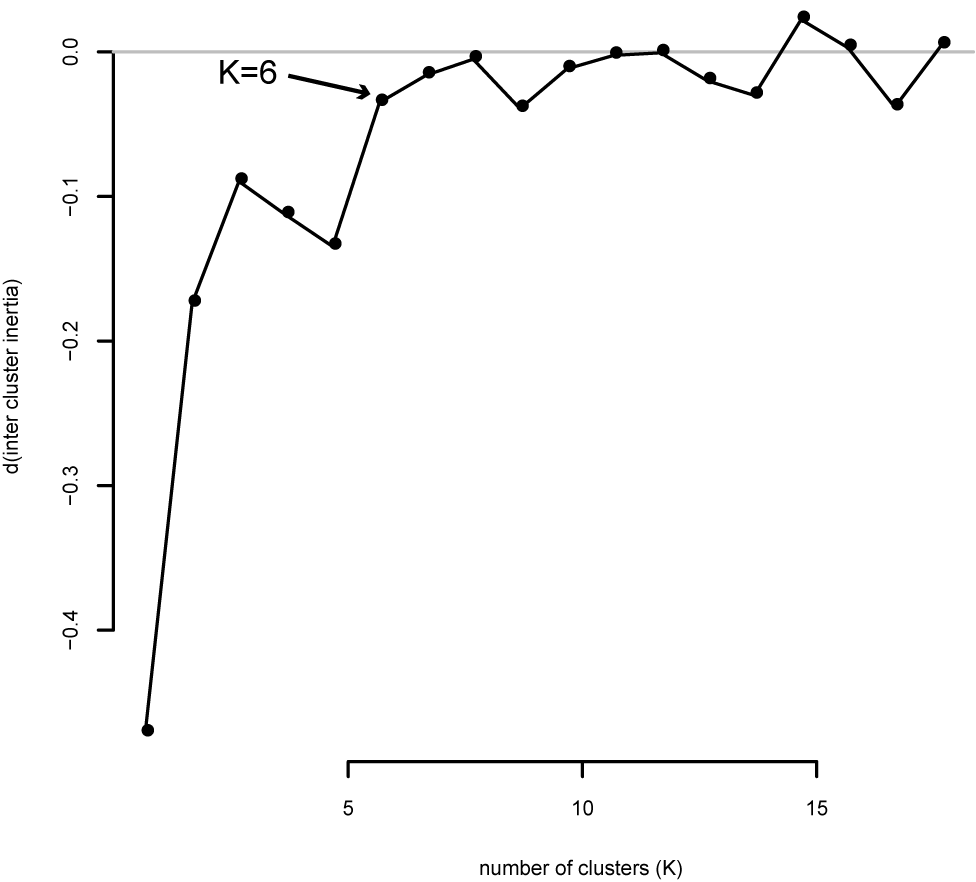

Supplement: Figure S1 — Inertia computed following Kergoat & Alvarez [34] , for each K value in the K-means computations on the plant AFLP dataset. (DOC) [file pone.0028662.s001.doc]

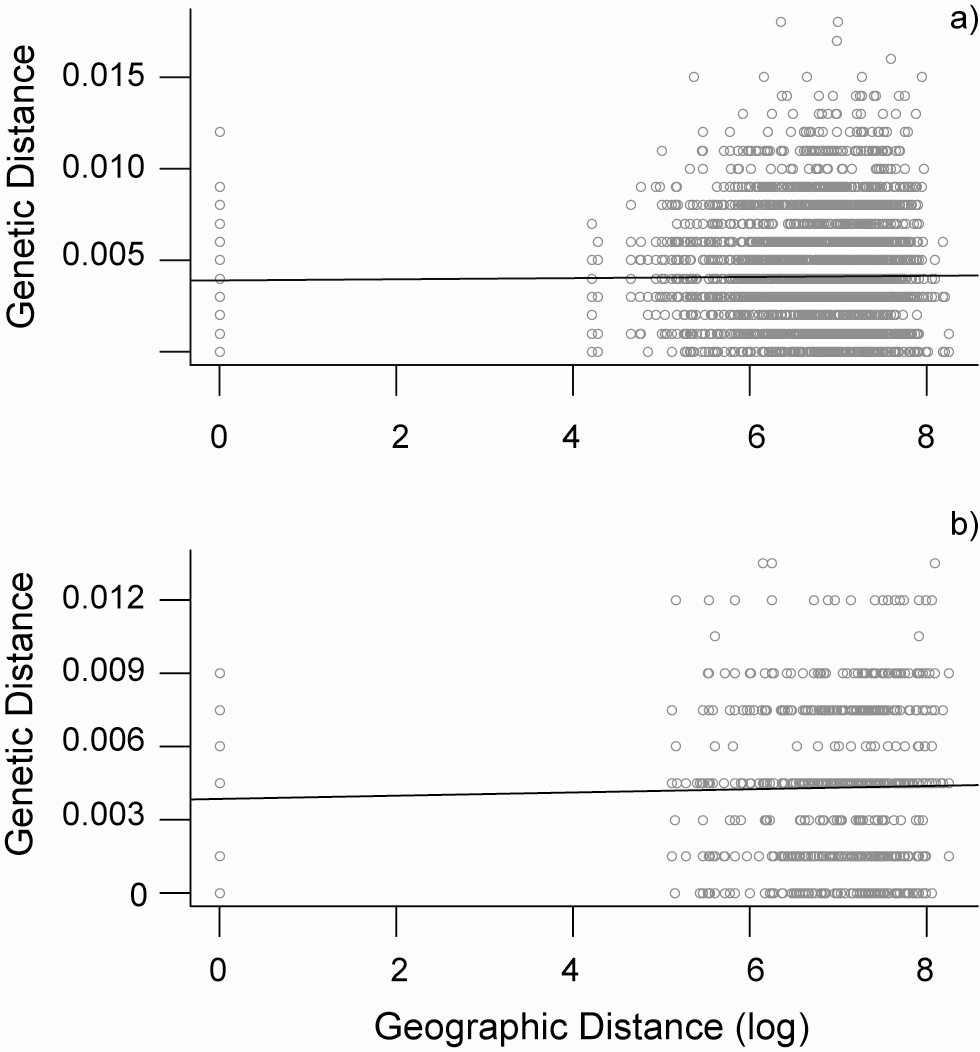

Supplement: Figure S3 — Isolation by distance for A) Psychoda phalaenoides and B) Psycha grisescens represented by the correlation between log geographic distances and genetic distances. Regression lines are shown. R2 are 0.002 and 0.006 for P. phalaenoides and P. grisescens, respectively, with P values>0.05 in both cases. (DOC) [file pone.0028662.s003.doc]
