# Supplementary material for: Comparative Phylogeography in a Specific and Obligate Pollination Antagonism
Source: PLoS One. 2011 Dec 27;6(12):e28662. doi: 10.1371/journal.pone.0028662 (PMC3246438; doi:10.1371/journal.pone.0028662)
Supplement: Figure S2 — Phylogeny of the two Psychodid species inferred applying Bayesian and Maximum Likelihood approaches. Support values are shown on branches, Bayesian/Maximum Likelihood. (DOC) [file pone.0028662.s002.doc]

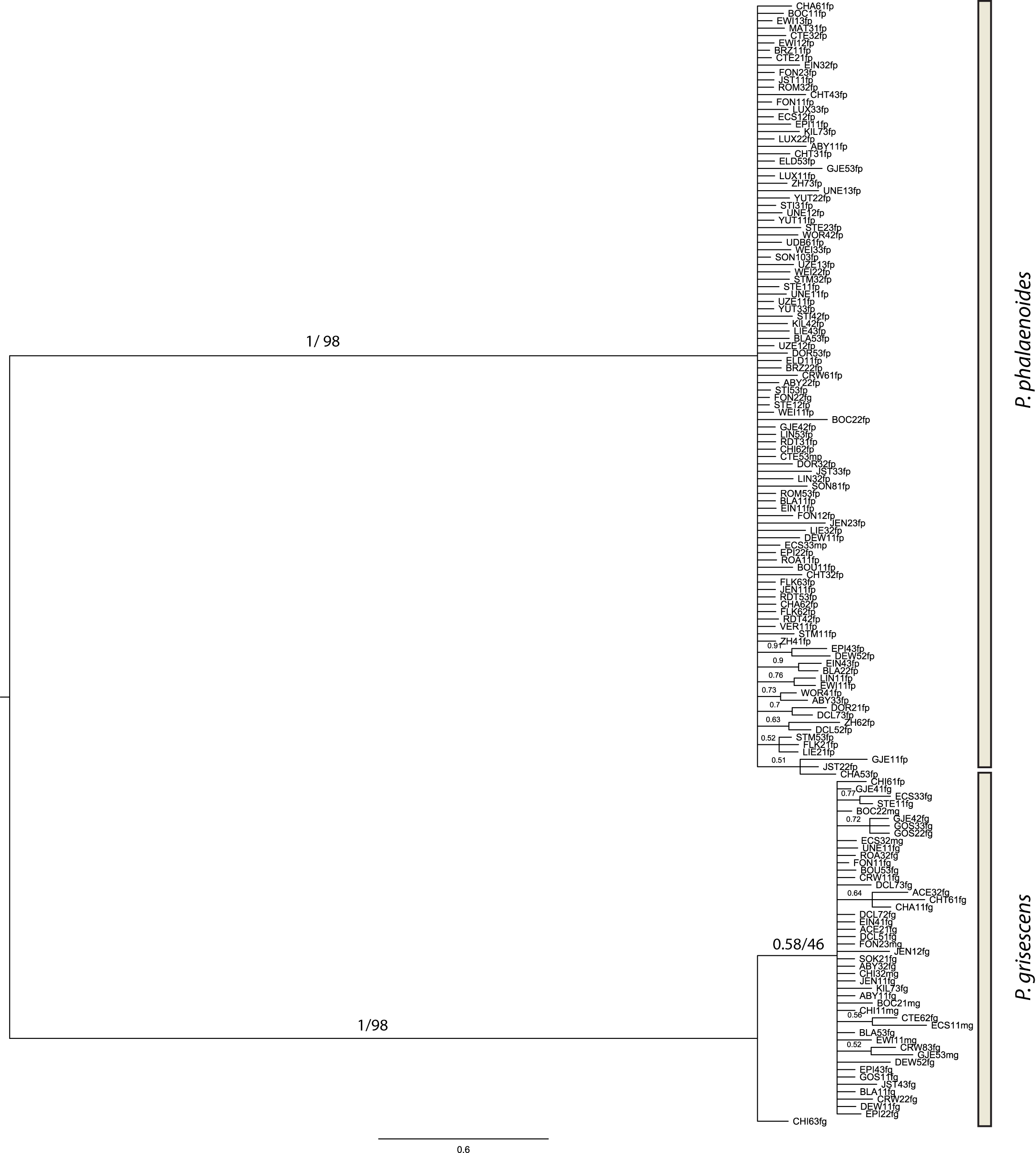


Both species are clearly separated, but no intraspecific structure is observed in any of the species.
