# Supplementary material for: Comparative Phylogeography in a Specific and Obligate Pollination Antagonism
Source: PLoS One. 2011 Dec 27;6(12):e28662. doi: 10.1371/journal.pone.0028662 (PMC3246438; doi:10.1371/journal.pone.0028662)
Supplement: Table S1 — Sampled locations with geographic coordinates. Number of plants, Psychoda phalaenoides and Psycha grisescens analyzed at each sampled location are given. Assignments to groups for the AMOVA tests are indicated under “group AMOVA”. (DOC) [file pone.0028662.s004.doc]

Table S1 – Sampled locations with geographic coordinates. Number of plants, *Psychoda phalaenoides* and *Psycha grisescens* analyzed at each sampled location are given. Assignment to groups for the AMOVA tests are indicated under “group AMOVA”.

| Location | Code | Longitude | Latitude | Elevation (m) | *A. maculatum* | | *P. grisescens* | | | *P. phalaenoides* | | |
| --- | --- | --- | --- | --- | --- | --- | --- | --- | --- | --- | --- | --- |
|  |  |  |  |  | # samples | group AMOVA | # samples | group AMOVA_strc_ | group AMOVA  _K-means_ | # samples | group AMOVA_strc_ | group AMOVA  _K-means_ |
| Aby | ABY | 10.16801 | 56.14328 | 0 | 5 | 1 | 2 | 1 | 5 | 3 | 1 | 5 |
| Acerno | ACE | 15.16854 | 40.77018 | 928 | 5 | 2 | 2 | 2 | 2 |  |  |  |
| Avala | AVA | 20.51481 | 44.69189 | 468 | 5 |  |  |  |  |  |  |  |
| Baile Herkulane | BHK | 22.4586 | 44.93176 | 300 | 5 |  |  |  |  |  |  |  |
| Balzers | LIE | 9.886944 | 47.15111 | 494 | 4 | 1 |  |  |  | 3 | 1 | 6 |
| Bausen | BAU | 0.7239 | 42.82485 | 610 | 5 |  |  |  |  |  |  |  |
| Blankenheim | BLA | 7.098333 | 50.73389 | 531 | 5 | 1 | 2 | 1 | 4 | 3 | 1 | 4 |
| Boc | BOC | 13.06557 | 50.34522 | 364 | 5 | 1 | 2 | 1 | 6 | 2 | 1 | 6 |
| Bois St. Pierre | BSP | 3.71207 | 50.29306 | 130 | 5 |  |  |  |  |  |  |  |
| Bouloire | BOU | 0.56135 | 47.97096 | 115 | 5 | 2 | 1 | 1 | 3 | 1 | 1 | 3 |
| Brzeg | BRZ | 17.4509 | 50.85675 | 336 | 5 | 1 |  |  |  | 2 | 1 | 6 |
| Chaumont | CHA | 5.09475 | 48.11508 | 296 | 5 | 1 | 1 | 1 | 3 | 3 | 1 | 3 |
| Chene-Bourg | GE | 6.18533 | 46.192782 | 430 | 2 |  |  |  |  |  |  |  |
| Chiflik | CHI | 24.52836 | 42.813 | 786 | 5 | 2 | 3 | 2 | 1 | 1 | 2 | 1 |
| Churchtown | CHT | -2.78686 | 53.88121 | 3 | 5 | 1 | 1 | 1 | 3 | 3 | 1 | 3 |
| Col de Roanza | ROA | 12.20989 | 46.17638 | 816 | 5 | 2 |  |  |  | 1 | 1 | 2 |
| Conteville | CTE | 1.73872 | 50.73731 | 60 | 5 | 1 | 1 | 1 | 3 | 3 | 1 | 3 |
| Cranwich | CRW | 0.60818 | 52.52699 | 24 | 5 | 2 | 3 | 1 | 3 | 1 | 1 | 3 |
| Dewley | DEW | -2.2918 | 52.38652 | 35 | 5 | 2 | 2 | 1 | 3 | 2 | 1 | 3 |
| Dornum | DOR | 7.43117 | 53.65095 | 0 | 5 | 1 |  |  |  | 3 | 1 | 5 |
| Duclair | DCL | 0.92016 | 49.48517 | -1 | 5 | 2 | 3 | 1 | 3 | 2 | 1 | 3 |
| Ecausseville | ECS | -1.37439 | 49.46218 | 0 | 5 | 1 | 3 | 1 | 3 | 2 | 1 | 3 |
| Ecublens | VD | 6.56637 | 46.52655 | 400 | 2 |  |  |  |  |  |  |  |
| Einhaus | EIN | 10.73472 | 53.71355 | 21 | 5 | 1 | 1 | 1 | 3 | 3 | 1 | 3 |
| Eldgasen | ELD | 9.62534 | 52.17533 | 101 | 5 | 1 |  |  |  | 2 | 1 | 3 |
| Epinal | EPI | 6.654722 | 48.45028 | 400 | 5 | 1 | 2 | 1 | 6 | 3 | 1 | 6 |
| Ewijk | EWI | 5.74856 | 51.8763 | 21 | 5 | 1 | 1 | 1 | 6 | 3 | 1 | 6 |
| Falkstone | FLK | 1.19223 | 51.09506 | 78 | 5 | 1 |  |  |  | 3 | 1 | 3 |
| Font de la Salut | FON | 2.49728 | 42.06183 | 1100 | 5 | 2 | 3 | 1 | 6 | 3 | 1 | 6 |
| Forêt d'Orient | FOR | 0.56135 | 48.1518 | 295 | 5 |  |  |  |  |  |  |  |
| Gabrovnitza | GAB | 25.17856 | 42.7295 | 731 | 4 |  |  |  |  |  |  |  |
| Genos | GEN | 0.40551 | 42.81207 | 975 | 5 |  |  |  |  |  |  |  |
| Gjeddesdal | GJE | 12.23064 | 55.60755 | 32 | 5 | 1 | 3 | 1 | 5 | 3 | 1 | 5 |
| Gostilje | GOS | 19.83549 | 43.65561 | 785 | 5 | 2 | 3 | 2 | 1 |  |  |  |
| Gross Disnack | GDI | 10.70769 | 53.72313 | 42 | 13 |  |  |  |  |  |  |  |
| Hohenweiler | LIN | 10.315556 | 48.00694 | 486 | 5 | 1 |  |  |  | 3 | 1 | 3 |
| Hönggerberg | ZH | 8.56637 | 47.40812 | 435 | 5 | 1 |  |  |  | 3 | 1 | 4 |
| Jenne | JEN | 13.18219 | 41.88142 | 824 | 6 | 1 | 2 | 2 | 2 | 2 | 2 | 2 |
| Johnstown Castle | JST | -6.50412 | 52.29245 | -18 | 5 | 1 | 1 | 1 | 3 | 3 | 1 | 3 |
| Kamena Gora | KAG | 19.56567 | 43.2983 | 1201 | 5 |  |  |  |  |  |  |  |
| Killaloe | KIL | -8.44438 | 52.7946 | 44 | 5 | 1 | 1 | 1 | 3 | 2 | 1 | 3 |
| La Groutte | GRO | 2.51343 | 46.69078 | 137 | 5 |  |  |  |  |  |  |  |
| La Loubatière | LOU | 2.2568 | 43.40768 | 720 | 4 |  |  |  |  |  |  |  |
| La Mignonnais | MSG | -1.86466 | 47.55066 | 16 | 3 |  |  |  |  |  |  |  |
| Laccio | LAC | 9.13009 | 44.48817 | 629 | 4 |  |  |  |  |  |  |  |
| Lago Matese | MAT | 14.40561 | 41.40639 | 1098 | 10 | 2 |  |  |  | 1 | 2 | 2 |
| Les | LES | 0.7237 | 42.82462 | 610 | 5 |  |  |  |  |  |  |  |
| Lescun | LEC | -0.6709 | 42.91148 | 1000 | 5 |  |  |  |  |  |  |  |
| Maiche | MAI | 7.339722 | 47.485 | 613 | 4 |  |  |  |  |  |  |  |
| Monte Pizi | MPI | 14.15127 | 41.92002 | 925 | 3 |  |  |  |  |  |  |  |
| Montese | MON | 10.98371 | 44.25523 | 707 | 5 |  |  |  |  |  |  |  |
| Morro Reatino | MOR | 12.85742 | 42.53585 | 759 | 6 |  |  |  |  |  |  |  |
| Quilen | QUI | 1.91746 | 50.52071 | 112 | 5 |  |  |  |  |  |  |  |
| Randalstown | RDT | -6.28349 | 54.79447 | 58 | 5 | 1 |  |  |  | 3 | 1 | 6 |
| Rifreddo | RIF | 15.82473 | 40.57235 | 1172 | 5 |  |  |  |  |  |  |  |
| Romsey | ROM | -1.52498 | 50.96424 | 52 | 5 | 1 |  |  |  | 2 | 1 | 3 |
| Sankt Margrethen | STM | 11.77574 | 47.3825 | 527 | 5 | 1 |  |  |  | 3 | 1 | 3 |
| Schmilau | SMI | 10.7575 | 53.66833 | 2 | 5 |  |  |  |  |  |  |  |
| Sitno | SIT | 14.84701 | 53.03406 | 66 | 5 | 1 |  |  |  | 3 | 1 | 5 |
| Sokobanja Vrh source | SOK | 21.88755 | 43.60373 | 844 | 10 | 2 | 1 | 2 | 1 |  |  |  |
| Sonderborg | SON | 9.82699 | 54.91554 | 10 | 7 | 1 |  |  |  | 2 | 1 | 5 |
| St. Fargeau | SFG | 3.09205 | 47.62233 | 226 | 5 |  |  |  |  |  |  |  |
| Steeg | STE | 13.63328 | 47.61349 | 513 | 5 | 1 | 1 | 1 | 6 | 3 | 1 | 6 |
| Suva Planina | SUV | 22.13166 | 43.21021 | 1114 | 4 |  |  |  |  |  |  |  |
| Tintesmühle | LUX | 6.223889 | 50.15611 | 305 | 4 | 1 |  |  |  | 3 | 1 | 5 |
| Udbina | UDB | 15.76134 | 44.53128 | 810 | 6 | 2 |  |  |  | 1 | 2 | 2 |
| Unec | UNE | 14.28609 | 45.82845 | 525 | 5 | 2 |  |  |  | 3 | 2 | 2 |
| Uzer | VZE | 0.17868 | 43.07732 | 580 | 5 | 1 |  |  |  | 3 | 1 | 3 |
| Verne | VER | 5.1905 | 45.22675 | 502 | 5 | 1 |  |  |  | 1 | 1 | 5 |
| Weilburg | WEI | 8.475556 | 50.88361 | 158 | 3 | 1 |  |  |  | 3 | 1 | 5 |
| Worbis | WOR | 10.38198 | 51.42088 | 370 | 4 | 1 |  |  |  | 2 | 1 | 3 |
| Yutz | YUT | 6.303889 | 49.55139 | 225 | 4 | 1 |  |  |  | 3 | 1 | 3 |
