# Supplementary material for: Comparative Phylogeography in a Specific and Obligate Pollination Antagonism
Source: PLoS One. 2011 Dec 27;6(12):e28662. doi: 10.1371/journal.pone.0028662 (PMC3246438; doi:10.1371/journal.pone.0028662)
Supplement: Table S2 — GenBank accession numbers and TreeBase link to data (**; URL: http://purl.org/phylo/treebase/phylows/study/TB2:S12057 ) of sequences used in this work. (DOC) [file pone.0028662.s005.doc]

Table S2 – GenBank accession numbers and TreeBase link to data (indicated as **; URL: <http://purl.org/phylo/treebase/phylows/study/TB2:S12057>) of sequences used in this work.

| **Sample** | **Species** | **Region** | | |
| --- | --- | --- | --- | --- |
|  |  | **cytB** | **16s** | **COI** |
| ABY11fg | *P. grisescens* | JN899752 | [**](http://purl.org/phylo/treebase/phylows/study/TB2:S12057) | - |
| ABY11fp | *P. phalaenoides* | JN899703 | - | - |
| ABY22fp | *P. phalaenoides* | JN899691 | - | JN899786 |
| ABY32fg | *P. grisescens* | JN899743 | - | - |
| ABY33fp | *P. phalaenoides* | JN899682 | - | JN899787 |
| ACE21fg | *P. grisescens* | JN899733 | - | - |
| ACE32fg | *P. grisescens* | JN899723 | - | - |
| BLA11fg | *P. grisescens* | JN899780 | - | - |
| BLA11fp | *P. phalaenoides* | JN899674 | - | - |
| BLA22fp | *P. phalaenoides* | JN899665 | - | - |
| BLA53fg | *P. grisescens* | JN899763 | JN899605 | - |
| BLA53fp | *P. phalaenoides* | JN899656 | [**](http://purl.org/phylo/treebase/phylows/study/TB2:S12057) | - |
| BOC11fp | *P. phalaenoides* | JN899645 | JN899606 | - |
| BOC21mg | *P. grisescens* | JN899753 | - | - |
| BOC22fp | *P. phalaenoides* | JN899638 | - | - |
| BOC22mg | *P. grisescens* | JN899744 | - | [**](http://purl.org/phylo/treebase/phylows/study/TB2:S12057) |
| BOU11fp | *P. phalaenoides* | JN899692 | [**](http://purl.org/phylo/treebase/phylows/study/TB2:S12057) | - |
| BOU53fg | *P. grisescens* | JN899716 | - | - |
| BRZ11fp | *P. phalaenoides* | JN899666 | - | JN899788 |
| BRZ22fp | *P. phalaenoides* | JN899657 | - | JN899789 |
| CHA11fg | *P. grisescens* | JN899781 | - | - |
| CHA53fp | *P. phalaenoides* | JN899693 | - | - |
| CHA61fp | *P. phalaenoides* | JN899639 | JN899607 | - |
| CHA62fp | *P. phalaenoides* | JN899704 | - | - |
| CHI11mg | *P. grisescens* | JN899754 | - | - |
| CHI32mg | *P. grisescens* | JN899745 | - | - |
| CHI61fp | *P. phalaenoides* | - | JN899608 | - |
| CHI62fp | *P. phalaenoides* | JN899646 | - | - |
| CHI63fg | *P. grisescens* | JN899734 | - | - |
| CHT31fp | *P. phalaenoides* | JN899705 | [**](http://purl.org/phylo/treebase/phylows/study/TB2:S12057) | - |
| CHT32fp | *P. phalaenoides* | JN899694 | - | - |
| CHT43fp | *P. phalaenoides* | JN899683 | [**](http://purl.org/phylo/treebase/phylows/study/TB2:S12057) | - |
| CHT61fg | *P. grisescens* | JN899724 | - | - |
| CRW11fg | *P. grisescens* | JN899717 | - | - |
| CRW22fg | *P. grisescens* | JN899782 | - | - |
| CRW61fp | *P. phalaenoides* | JN899675 | - | JN899790 |
| CRW83fg | *P. grisescens* | JN899771 | - | - |
| CTE21fp | *P. phalaenoides* | JN899667 | [**](http://purl.org/phylo/treebase/phylows/study/TB2:S12057) | [**](http://purl.org/phylo/treebase/phylows/study/TB2:S12057) |
| CTE32fp | *P. phalaenoides* | JN899658 | JN899609 | - |
| CTE53mp | *P. phalaenoides* | JN899647 | - | - |
| CTE62fg | *P. grisescens* | JN899755 | - | - |
| DCL51fg | *P. grisescens* | JN899735 | - | - |
| DCL52fp | *P. phalaenoides* | JN899706 | - | - |
| DCL72fg | *P. grisescens* | JN899725 | - | - |
| DCL73fg | *P. grisescens* | JN899718 | - | - |
| DCL73fp | *P. phalaenoides* | JN899695 | - | - |
| DEW11fg | *P. grisescens* | JN899783 | - | - |
| DEW11fp | *P. phalaenoides* | JN899684 | - | - |
| DEW52fg | *P. grisescens* | JN899772 | - | - |
| DEW52fp | *P. phalaenoides* | JN899676 | - | - |
| DOR21fp | *P. phalaenoides* | JN899659 | - | - |
| DOR32fp | *P. phalaenoides* | JN899648 | - | - |
| DOR53fp | *P. phalaenoides* | JN899640 | - | JN899791 |
| ECS11mg | *P. grisescens* | JN899756 | - | - |
| ECS12fp | *P. phalaenoides* | JN899696 | JN899610 | - |
| ECS32mg | *P. grisescens* | JN899746 | - | JN899792 |
| ECS33fg | *P. grisescens* | JN899736 | - | [**](http://purl.org/phylo/treebase/phylows/study/TB2:S12057) |
| ECS33mp | *P. phalaenoides* | JN899685 | - | - |
| EIN11fp | *P. phalaenoides* | JN899677 | - | - |
| EIN32fp | *P. phalaenoides* | JN899668 | [**](http://purl.org/phylo/treebase/phylows/study/TB2:S12057) | JN899793 |
| EIN41fg | *P. grisescens* | JN899726 | - | - |
| EIN43fp | *P. phalaenoides* | JN899660 | JN899611 | - |
| ELD11fp | *P. phalaenoides* | JN899649 | - | JN899794 |
| ELD53fp | *P. phalaenoides* | JN899707 | [**](http://purl.org/phylo/treebase/phylows/study/TB2:S12057) | - |
| EPI11fp | *P. phalaenoides* | JN899697 | [**](http://purl.org/phylo/treebase/phylows/study/TB2:S12057) | - |
| EPI22fg | *P. grisescens* | JN899784 | - | - |
| EPI22fp | *P. phalaenoides* | JN899686 | - | - |
| EPI43fg | *P. grisescens* | JN899773 | [**](http://purl.org/phylo/treebase/phylows/study/TB2:S12057) | - |
| EPI43fp | *P. phalaenoides* | JN899678 | - | - |
| EWI11fp | *P. phalaenoides* | JN899669 | - | - |
| EWI11mg | *P. grisescens* | JN899764 | - | - |
| EWI12fp | *P. phalaenoides* | JN899661 | [**](http://purl.org/phylo/treebase/phylows/study/TB2:S12057) | - |
| EWI13fp | *P. phalaenoides* | JN899650 | JN899612 | [**](http://purl.org/phylo/treebase/phylows/study/TB2:S12057) |
| FLK21fp | *P. phalaenoides* | JN899641 | - | - |
| FLK62fp | *P. phalaenoides* | JN899708 | - | - |
| FLK63fp | *P. phalaenoides* | JN899698 | - | - |
| FON11fg | *P. grisescens* | JN899757 | - | JN899813 |
| FON11fp | *P. phalaenoides* | JN899687 | [**](http://purl.org/phylo/treebase/phylows/study/TB2:S12057) | JN899795 |
| FON12fp | *P. phalaenoides* | JN899679 | - | - |
| FON22fg | *P. grisescens* | JN899747 | - | JN899796 |
| FON23fp | *P. phalaenoides* | JN899670 | [**](http://purl.org/phylo/treebase/phylows/study/TB2:S12057) | - |
| FON23mg | *P. grisescens* | JN899737 | - | - |
| GJE11fp | *P. phalaenoides* | JN899651 | [**](http://purl.org/phylo/treebase/phylows/study/TB2:S12057) | JN899797 |
| GJE41fg | *P. grisescens* | JN899727 | - | JN899798 |
| GJE42fg | *P. grisescens* | JN899719 | - | JN899799 |
| GJE42fp | *P. phalaenoides* | JN899642 | - | - |
| GJE53fp | *P. phalaenoides* | JN899709 | [**](http://purl.org/phylo/treebase/phylows/study/TB2:S12057) | - |
| GJE53mg | *P. grisescens* | JN899785 | - | - |
| GOS11fg | *P. grisescens* | JN899774 | - | - |
| GOS22fg | *P. grisescens* | JN899765 | - | - |
| GOS33fg | *P. grisescens* | JN899758 | - | - |
| JEN11fg | *P. grisescens* | JN899748 | - | - |
| JEN11fp | *P. phalaenoides* | JN899699 | - | - |
| JEN12fg | *P. grisescens* | JN899738 | - | - |
| JEN23fp | *P. phalaenoides* | JN899680 | - | - |
| JST11fp | *P. phalaenoides* | JN899671 | [**](http://purl.org/phylo/treebase/phylows/study/TB2:S12057) | - |
| JST22fp | *P. phalaenoides* | JN899662 | - | - |
| JST33fp | *P. phalaenoides* | JN899652 | - | - |
| JST43fg | *P. grisescens* | JN899775 | - | - |
| KIL42fp | *P. phalaenoides* | JN899710 | JN899613 | - |
| KIL73fg | *P. grisescens* | JN899749 | [**](http://purl.org/phylo/treebase/phylows/study/TB2:S12057) | - |
| KIL73fp | *P. phalaenoides* | JN899700 | - | - |
| LIE21fp | *P. phalaenoides* | JN899688 | - | - |
| LIE32fp | *P. phalaenoides* | JN899681 | - | - |
| LIE43fp | *P. phalaenoides* | JN899672 | JN899614 | - |
| LIN11fp | *P. phalaenoides* | JN899663 | - | - |
| LIN32fp | *P. phalaenoides* | JN899653 | - | - |
| LIN53fp | *P. phalaenoides* | JN899643 | - | - |
| LUX11fp | *P. phalaenoides* | JN899711 | [**](http://purl.org/phylo/treebase/phylows/study/TB2:S12057) | - |
| LUX22fp | *P. phalaenoides* | JN899701 | JN899615 | - |
| LUX33fp | *P. phalaenoides* | JN899689 | [**](http://purl.org/phylo/treebase/phylows/study/TB2:S12057) | - |
| MAT31fp | *P. phalaenoides* | JN899654 | JN899616 | JN899800 |
| RDT31fp | *P. phalaenoides* | JN899644 | - | - |
| RDT42fp | *P. phalaenoides* | JN899712 | - | - |
| RDT53fp | *P. phalaenoides* | JN899702 | - | - |
| ROA11fp | *P. phalaenoides* | JN899690 | - | - |
| ROA32fg | *P. grisescens* | - | - | JN899815 |
| ROM32fp | *P. phalaenoides* | JN899673 | JN899617 | - |
| ROM53fp | *P. phalaenoides* | JN899664 | - | - |
| SOK21fg | *P. grisescens* | JN899739 | - | - |
| SON103fp | *P. phalaenoides* | JN899766 | JN899618 | JN899801 |
| SON81fp | *P. phalaenoides* | JN899655 | - | - |
| STE11fg | *P. grisescens* | JN899728 | - | JN899814 |
| STE11fp | *P. phalaenoides* | JN899759 | [**](http://purl.org/phylo/treebase/phylows/study/TB2:S12057) | JN899802 |
| STE12fp | *P. phalaenoides* | JN899750 | - | JN899803 |
| STE23fp | *P. phalaenoides* | JN899740 | [**](http://purl.org/phylo/treebase/phylows/study/TB2:S12057) | JN899804 |
| STI31fp | *P. phalaenoides* | JN899729 | JN899619 | JN899805 |
| STI42fp | *P. phalaenoides* | JN899720 | JN899620 | JN899806 |
| STI53fp | *P. phalaenoides* | JN899713 | - | JN899807 |
| STM11fp | *P. phalaenoides* | JN899776 | - | - |
| STM32fp | *P. phalaenoides* | JN899767 | JN899621 | - |
| STM53fp | *P. phalaenoides* | JN899760 | JN899622 | JN899808 |
| UDB61fp | *P. phalaenoides* | - | JN899623 | - |
| UNE11fg | *P. grisescens* | - | - | JN899809 |
| UNE11fp | *P. phalaenoides* | JN899741 | JN899624 | JN899810 |
| UNE12fp | *P. phalaenoides* | JN899730 | [**](http://purl.org/phylo/treebase/phylows/study/TB2:S12057) | JN899811 |
| UNE13fp | *P. phalaenoides* | JN899721 | JN899625 | JN899812 |
| UZE11fp | *P. phalaenoides* | JN899714 | JN899626 | - |
| UZE12fp | *P. phalaenoides* | JN899777 | JN899627 | - |
| UZE13fp | *P. phalaenoides* | JN899768 | JN899628 | - |
| VER11fp | *P. phalaenoides* | JN899731 | - | - |
| WEI11fp | *P. phalaenoides* | JN899778 | - | [**](http://purl.org/phylo/treebase/phylows/study/TB2:S12057) |
| WEI22fp | *P. phalaenoides* | JN899769 | JN899629 | - |
| WEI33fp | *P. phalaenoides* | JN899761 | JN899630 | - |
| WOR41fp | *P. phalaenoides* | JN899751 | JN899631 | [**](http://purl.org/phylo/treebase/phylows/study/TB2:S12057) |
| WOR42fp | *P. phalaenoides* | JN899742 | JN899632 | - |
| YUT11fp | *P. phalaenoides* | JN899732 | JN899633 | - |
| YUT22fp | *P. phalaenoides* | JN899722 | JN899634 | - |
| YUT33fp | *P. phalaenoides* | JN899715 | JN899635 | - |
| ZH41fp | *P. phalaenoides* | JN899779 | - | - |
| ZH62fp | *P. phalaenoides* | JN899770 | JN899636 | - |
| ZH73fp | *P. phalaenoides* | JN899762 | JN899637 | - |
